# Uncommon Case of Oral Vancomycin Neurotoxicity With Sexual Dysfunction

**DOI:** 10.7759/cureus.12396

**Published:** 2020-12-31

**Authors:** Ahmed Almohammadi, Omar Shahada, Abdulhadi Z Almadani, Muath Alqayidi

**Affiliations:** 1 Internal Medicine, Taibah University, Medina, SAU; 2 Dermatology, Taibah University, Medina, SAU; 3 Medicine, Taibah University, Medina, SAU

**Keywords:** vancomycin, neurotoxicity, sexual dysfunction, clostridium difficile

## Abstract

We report a case of a 57-year-old male with a past medical history of controlled hypertension and type 2 diabetes mellitus, who had unusual elevated serum concentration levels of vancomycin during oral treatment for *Clostridium difficile* pseudomembranous colitis. Both measured serum vancomycin levels of 39 μg/ml and cerebrospinal fluid level of 5.7 μg/mL were documented and associated with unexplained neurological symptoms and sexual dysfunction. After discontinuing of oral vancomycin and treating with hemodialysis, the patient had reduced serum concentration levels to 26 μg/mL accompanied by significant and rapid resolution of symptoms in addition to the general condition. Patients with intestinal disease and reduced renal function who are being treated with oral vancomycin may absorb and accumulate high serum level amounts of vancomycin, causing uncommon neurological toxication symptoms including headache, altered state of consciousness, confusion, and drowsiness in association with sexual dysfunction.

## Introduction

*Clostridium difficile* infection (CDI) has become more common with the increasing extensive therapeutic use of antibiotics [1]. Studies suggest the possibility of inadequate oral vancomycin absorption from the digestive tract due to its molecular size and its pharmacokinetics [2]. It is known to have an approximate 5 to 11 hours of systemic elimination time in patients with no renal disorders or reduced renal function. However, previous reports show that a detectable serum concentration may be produced following an oral vancomycin therapy in patients with renal failure and severe colitis [3,4]. We present an uncommon case of vancomycin neurotoxicity with sexual dysfunction following an oral treatment for *Clostridium difficile* pseudomembranous colitis.

## Case presentation

A 57-year-old male with a past medical history of hypertension and type 2 diabetes mellitus controlled with enalapril and lifestyle modifications presented to the emergency department complaining of diarrhea started six days ago with foul odor stool and associated mild abdominal pain. He was recently treated with clindamycin for respiratory tract infection. On examination, the patient was conscious, oriented, and vitally stable, abdominal palpation revealed mild tenderness, and no other significant findings on the rest of the examination. A diagnosis of CDI was confirmed using stool polymerase chain reaction (PCR) test, which was positive for toxin B; other lab tests were unremarkable, including creatinine levels and negative cerebrospinal fluid (CSF) gram stain culture. The patient was treated conservatively at the emergency department and discharged with oral vancomycin 125 mg every six hours with follow-up in the out-patient department. After 14 days, the patient complained of a headache and drowsiness associated with sexual dysfunction that kept worsening. On examination, he had altered state of consciousness and appeared confused with a Glasgow coma scale score of 11; the rest of the examination was unremarkable. On laboratory tests, both serum vancomycin levels of 39 μg/mL and CSF level of 5.7 μg/ml were documented. The vancomycin dose was reduced to 125 mg every 12 hours, with a regular follow-up. Three days later, the patient reported mild improvement but with persistent symptoms. Oral vancomycin was stopped, and hemodialysis was started with gradual improvement and disappearance of symptoms overtime. On day 6 post-discontinuation of the drug, after 24 hours of the latest dose detectable at 2.2 mcg/dL, a random serum vancomycin level test was conducted, which resulted undetectable. Resolution of symptoms after stopping oral treatment and initiating hemodialysis, as well as a detectable serum level of vancomycin reflect systemic accumulation with subsequent toxicity.

## Discussion

CDI is a common etiology of nosocomial diarrhea in hospitals and other long-term care facilities, resulting in increased burden of financial expense [1]. CDI is currently seen as a significant community pathogen due to its epidemiology increase in the previous decade [1,5]. Approximately one out of four patients had repeated infection [6]. It has become evident that vancomycin is more effective than other antibiotics, including metronidazole, when treating CDI [7]. The treatment guidelines for oral vancomycin recommend its use for severe cases and after the first infection recurrence [8]. The poor systemic absorption of oral vancomycin predicted by its pharmacokinetics character forms the basis for use of oral vancomycin for colitis due to CD [3]. However, systemic absorption after administration of oral vancomycin had conflicting clinical data. Some documented cases showed serum vancomycin detectable levels after administration of oral vancomycin in cases of severe colitis and in patients with reduced renal function [3,9]. In a series of 10 reported cases taking therapeutic oral vancomycin, 4 out of 10 of these cases had a detectable serum vancomycin level ranging from 1.0 to 3.1 mg/L. In these four cases, one patient had reduced renal function [10]. Furthermore, an observational study with 85 patients observed a detectable level of serum vancomycin in 68% of patients. Other observed and documented risk factors for systemic exposure of oral vancomycin included intensive-care unit admissions, reduced renal function, active severe CDI, over 10 days of therapy, inflammatory disorders of the gastrointestinal tract, and associated vancomycin retention enema usage [4]. Some previous reports suggest the lack of oral vancomycin systemic absorption [11]. A late pilot study including eight children, in which seven children had a diagnosis of inflammatory bowel disease and one child with acute kidney injury, observed no detectable levels of serum vancomycin following oral vancomycin [12]. A prospective study including 57 adults revealed no detectable levels of serum vancomycin in 98% of the cases taking oral vancomycin treatment. Moreover, it is known that the major route of vancomycin systemic clearance is processed by renal excretion, and no occurrence of a proper systemic absorption was noted including in cases with reduced renal function [13]. Permanent or transient neurotoxicity symptoms with sexual dysfunction have rarely been considered as side effects of oral vancomycin treatment and are linked to significant accumulation of vancomycin serum levels [2]. Typically, the symptoms subside after decreasing the dose or discontinuing oral vancomycin. There are limited reports of vancomycin toxicity, but none was reported with neurotoxicity in addition to sexual dysfunction. Our case is uncommon regardless of the absence of reduced renal function and known risk factors for systemic exposure to vancomycin; the patient presented with symptoms indicating neurotoxicity that may promote or mimic mild encephalitis. No concurrent known medication to cause neurotoxicity or possible drug interactions with vancomycin was taken by the patient. A measurement of vancomycin serum level of 2.2 mcg/dL has confirmed the systemic absorption about 24 hours after discontinuing the drug. Using the calculation method of the adverse drug reaction probability defined by Naranjo et al., our case would be considered a “probable” drug reaction due to oral vancomycin [14].

## Conclusions

Our case report shows a possibility of vancomycin accumulation in serum and eventually reaching neurotoxic levels regardless of the absence of renal impairment and other related known risk factors for systemic absorption. The likelihood of systemic absorption and possible vancomycin neurotoxicity should be considered and discussed with patients when prescribing oral vancomycin therapy.
